# Increasing throughput of AFM-based single cell adhesion measurements through multisubstrate surfaces

**DOI:** 10.3762/bjnano.6.15

**Published:** 2015-01-14

**Authors:** Miao Yu, Nico Strohmeyer, Jinghe Wang, Daniel J Müller, Jonne Helenius

**Affiliations:** 1 Department of Biosystems Science and Engineering, ETH Zurich, Mattenstrasse 26, 4058 Basel, Switzerland; 2 Center for Precision Engineering, Harbin Institute of Technology, Harbin 150001, China

**Keywords:** atomic force microscopy, cell adhesion, collagen I, fibroblasts, fibronectin, HeLa, laminin, MDCK, PC3, single cell assay, single cell force spectroscopy

## Abstract

Mammalian cells regulate adhesion by expressing and regulating a diverse array of cell adhesion molecules on their cell surfaces. Since different cell types express distinct sets of cell adhesion molecules, substrate-specific adhesion is cell type- and condition-dependent. Single-cell force spectroscopy is used to quantify the contribution of cell adhesion molecules to adhesion of cells to specific substrates at both the cell and single molecule level. However, the low throughput of single-cell adhesion experiments greatly limits the number of substrates that can be examined. In order to overcome this limitation, segmented polydimethylsiloxane (PDMS) masks were developed, allowing the measurement of cell adhesion to multiple substrates. To verify the utility of the masks, the adhesion of four different cell lines, HeLa (Kyoto), prostate cancer (PC), mouse kidney fibroblast and MDCK, to three extracellular matrix proteins, fibronectin, collagen I and laminin 332, was examined. The adhesion of each cell line to different matrix proteins was found to be distinct; no two cell lines adhered equally to each of the proteins. The PDMS masks improved the throughput limitation of single-cell force spectroscopy and allowed for experiments that previously were not feasible. Since the masks are economical and versatile, they can aid in the improvement of various assays.

## Introduction

The regulated adhesion of mammalian cells with the extracellular matrix (ECM) and surrounding cells is crucial in biological processes such as cell migration, differentiation, proliferation, and apoptosis. Since impaired cell adhesion causes a wide range of diseases, the study of cell adhesion is an important field of research [1–5]. Cell adhesion is predominantly mediated by cell adhesion molecules (CAMs), which comprise different protein families, including integrins and cadherins [6]. Cells express and regulate CAMs in order to control whether they adhere to surfaces they encounter, and if so, how strong and for how long [7–10]. Extracellular cues and intracellular signaling tightly regulate cell adhesion. Furthermore, the outside-in signaling of CAMs regulate cellular processes including the adhesive properties of the cell [11]. Among CAMs, integrins dominantly facilitate adhesion of cells to ECM proteins. Integrins are heterodimers composed of non-covalently linked α- and β-subunits, both of which consist of a large extracellular domain, a short transmembrane domain, and a cytoplasmic domain of variable length. In mammalian cells, the 18 α-subunits and 8 β-subunits are known to form 24 different integrins, which have specific, but overlapping, adhesion functions and often bind to more than one ECM protein [12]. To adapt their adhesion to the ECM, cells regulate the surface expression of integrins [13]. Importantly, cells differ in their adherence to various ECM proteins, necessitating the investigation of the adhesive properties of the cells.

**Figure 1:**
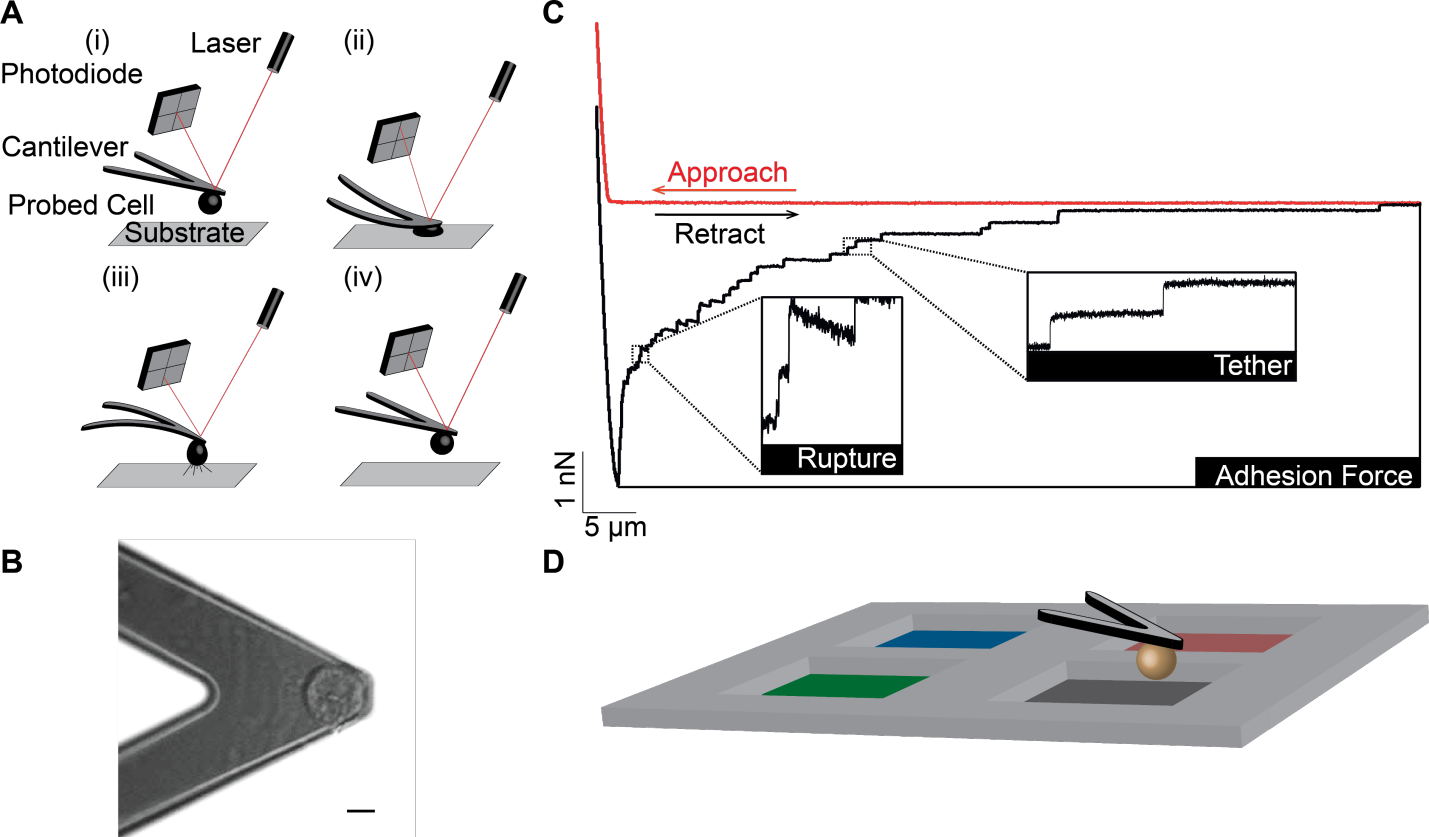
Depiction of AFM-based SCFS. (A,B) A single cell is bound to a tip-less AFM cantilever via a receptor specific or unspecific substrate (scale bar, 10 µm). (A) (i and ii) The cantilever-bound cell is brought into contact with the substrate until a preset force is recorded. After a specified contact time, (iii) the cantilever is retracted until the cell is fully detached from the substrate. During the experimental cycle, the deflection (force) of the cantilever and the distance between cell and surface is recorded in a force–distance curve (red). (C) The force–distance curve shows distinct features: In the approach segment of the force–distance curve, the deflection of the cantilever is recorded while the cell is lowered onto the substrate; the retraction segment of the force–distance curve (black) records the adhesion force of the cell, which is the maximum force acting on the cantilever and, thus, the force needed to initiate the detachment of the cell from its substrate. Subsequently, single receptor unbinding events are observed. Rupture events are recorded when the CAM–ligand bond of a cytoskeleton-linked CAM fails. Tether events are recorded when a membrane tether is extruded from the cell membrane with the CAM at its tip (tethers). In the latter case, attachment of the CAM to the cytoskeleton is either too weak to resist the mechanical stress applied or non-existent. (D) To improve the throughput of SCFS experiments, a four-segmented coating mask is used, allowing adhesion force measurements of one cell to different adhesives substrates.

Atomic force microscopy (AFM)-based single-cell force spectroscopy (SCFS) provides a versatile tool to quantify the adhesion of single cells in near-physiological conditions [14–16]. In AFM-based SCFS, a single cell is attached to a cantilever (Figure 1A,B), commonly facilitated by an adhesive coating (e.g., concanavalin A, poly-L-lysine or CellTak) [17–22]. The attached cell is lowered (approach) onto a substrate (Figure 1A(i)), which is a protein-coated surface, another cell or a biomaterial [23], until a set force is reached and the cell is kept stationary for a set time to allow the formation of adhesive interactions (Figure 1A(ii)). During the subsequent raising (retraction) of the cantilever (Figure 1A(iii)), the force acting on the cell and the distance between cell and substrate is recorded in a force–distance curve (Figure 1C). The force range that can be detected with AFM-based SCFS is from ≈10 pN up to ≈100 nN [14], thereby, SCFS allows both the overall cell adhesion and the contribution of single adhesion receptors to be quantified. During initial cantilever retraction the upward acting force on the cell increases until the force needed to initiate cell de-adhesion is reached, thereafter, unbinding events occur (Figure 1C). The maximum force is called the adhesion force and is a measure of how strong the cell adhered to the substrate. Unbinding events correlate with the unbinding of either single or clustered CAMs and can be characterized as either rupture or tether events [15,17,20]. The analysis of these unbinding events may be used to characterize the strength of single bonds and cell membrane properties [17,24–25]. Examples of the utility of SCFS include studies of the adhesion of two *Dictyostelium discoideum* cells via glycoproteins [26], dendritic cells via activated leukocyte cell adhesion molecules [17], Chinese hamster ovary cells to collagen I via α_2_β_1_-integrins [22], pre-osteoblasts to denatured collagen I via α_5_β_1_-integrins and integrins containing α_v_-subunits [27], Jurkat T cells to the vascular cell adhesion molecule VCAM-1 via α_4_β_1_-integrins [28], and the contribution of galectins to the overall cell adhesion of MDCK cells to collagens [29]. In addition to studying the adhesion of cells to a substrate, the regulation of one CAM by another CAM has been studied by SCFS. It was shown by SCFS that collagen I binding integrins down-regulate the avidity of fibronectin binding integrins by an increased endocytosis in HeLa cells [30].

The classical SCFS setup, where the adhesion of a cantilever-bound cell to a substrate is probed, has a limited throughput because only one substrate is examined per cantilever. Therefore, an alternative method has been used to quantify the adhesive properties of several cells using one cantilever. Thereto, in an inverted assay, a ligand-functionalized (e.g., ECM protein) cantilever or a cantilever with a functionalized bead is lowered on round or spread cells that are seeded on a Petri dish [31–32]. After the designated contact time has been reached, the cantilever is retracted from the cell and the adhesion of the cell to the cantilever or bead is measured. Thereafter, the cantilever can be moved above another cell and the adhesion experiment cycle repeated. With this inverted approach, several cells can be examined using one cantilever. However, a surface that has been in contact with a cell may be contaminated with debris from the cell or restructured by the cell, especially after longer contact times [16,33]. Due to the limited surface areas at the ends of the cantilevers and beads, the coating is compromised after a few measurements and the cantilever must be replaced to ensure consistent assay conditions.

An alternative method to increase the throughput of adhesion measurements in the classical setup is to microstructure the surface such that it presents areas having different properties. Examples used for SCFS include: microstructured surfaces with two different polymers [34], different nanoscale groves [35] and the use of two different ECM proteins [36]. However, the equipment needed for these approaches are uncommon in biological laboratories.

In order to increase the throughput, we chose to modify Petri dishes using polydimethylsiloxane (PDMS) masks with four distinct areas (Figure 1D). The four segments separate the Petri dish surface into four independent 4 × 4 mm^2^ wells, which allow one dish to be coated with different substrates and, thus, characterization of the adhesion of the same cell to four substrates. The masks are thin enough to remain on the Petri dish while performing adhesion measurements. These multi-segmented substrates not only increase the rate at which SCFS measurements can be made but also improve their reliability and comparability, because the adhesion of cells of the same type can vary considerably [37]. Therefore, the number of cells probed can be reduced. In addition, the masks decrease the coating area, reduce experimental material and improve experimental efficiency. We implemented the masks to characterize the adhesion of different cell lines to collagen I, fibronectin and laminin 332 and found that the cell lines had unique adhesion profiles, which likely reflect differences in the CAMs they expressed.

## Results

### PDMS masks for single-cell force spectroscopy

**Figure 2:**
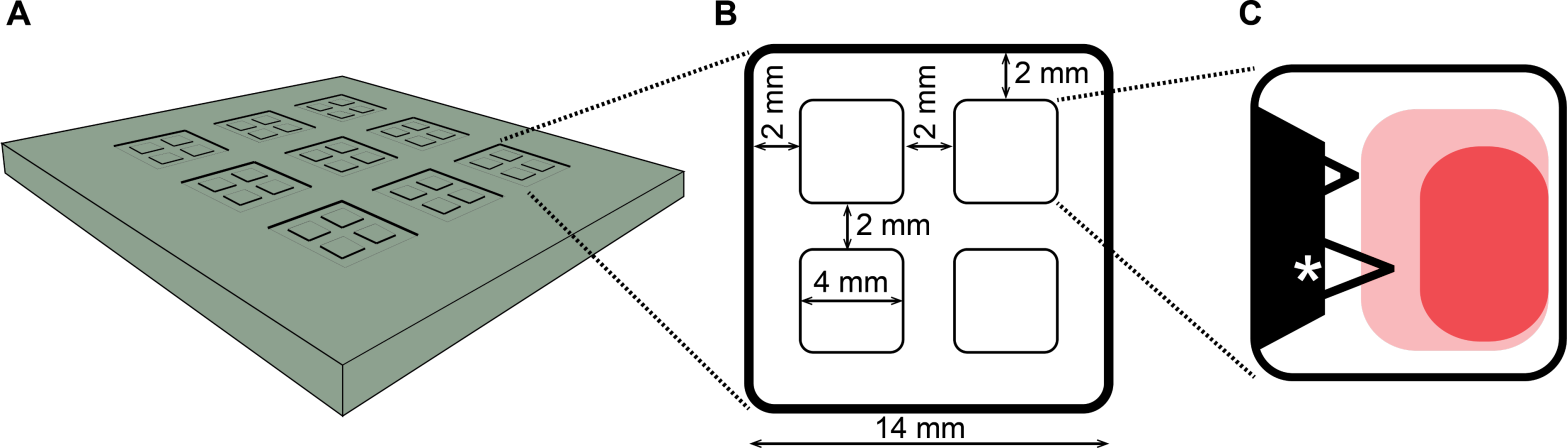
Technical details of the PDMS coating mask. (A) For the production of the PDMS masks, an aluminum mold is produced with nine casting molds for the PDMS masks. Dimensions of the masks are shown in (B). The height of the wells is 150 µm. (C) AFM cantilever (starred, NP-O, Bruker) accessible area usually used for SCFS adhesion experiments. Dark red indicates the area where the PDMS masks do not interfere with measurements, where the pale red area indicates regions where small interference occurs, and white areas are where measurements are not possible.

To increase throughput of AFM-based SCFS, we developed segmented PDMS masks (Figure 1D and Figure 2B) that allow the adhesion of one cell to different substrates to be characterized. Using this mask, four wells having an area of 16 mm^2^ and a depth of 150 µm (Figure 2B) can be coated in one Petri dish and SCFS adhesion measurements can be performed with the same cell in each segment without removing the mask. Since the non-specific adhesion of different cell lines to either glass or PDMS varies, we developed two types of PDMS masks with a protein coating on either a glass or PDMS surface. The mask type can be chosen to minimize the background adhesion of the cell line. The production process as well as the handling of the PDMS mask is described in the next sections. In addition, the characterization of surface topography, protein coating and microscopy utility of both types of PDMS masks is presented.

### Characterization of the protein coating on PDMS masks

**Figure 3:**
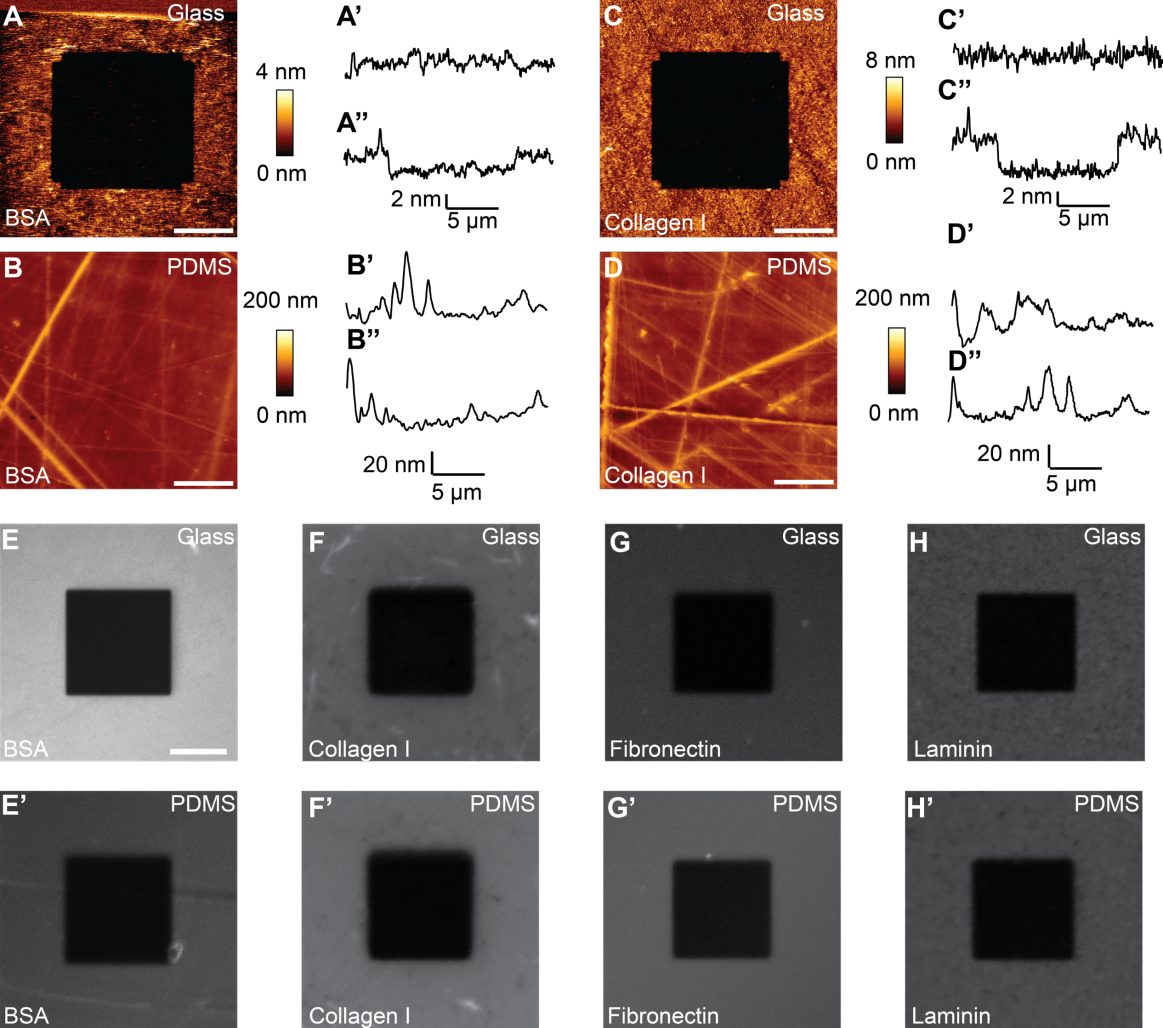
Surface characterization. Representative topographies of BSA-coated (A) and collagen I-coated (C) glass surfaces, and BSA-coated (B) and collagen I-coated (D) PDMS surfaces. A 20 × 20 µm^2^ area of the coated surface was imaged with AFM after a 10 x 10 µm^2^ area was repeatedly scratched by contact-mode AFM imaging using a high contact force. Images show a cavity in the scratched area for BSA- (A) and collagen I-coated (C) glass surfaces. The thicknesses of the coatings were similar, ≈3.5 nm for BSA (A’’) and ≈4.5 nm for collagen I (C’’). The PDMS surface showed a rough surface with height differences of >40 nm (B and D). On PDMS, the displacement of proteins by AFM scratching was not evident (B, B’’ and D, D’’). Scale bars, 5 µm. Since a protein coating was not demonstrated, we coated PDMS wells with fluorescently labeled BSA (FITC), collagen I (FITC), fibronectin (rhodamine) and laminin (HiLyte488) and imaged the glass (E–H, respectively) and PDMS (E’–F’) surfaces. Using confocal microscopy, a 20 × 20 µm^2^ area was bleached with maximum laser power before the 50 × 50 µm^2^ area was imaged (E–H and E’–H’). Scale bar, 10 µm.

To characterize the surface roughness and the protein coating, glass and PDMS surfaces were imaged using AFM. Several protein-coated surfaces of both PDMS and glass were imaged. Surfaces of protein-coated glass were smooth with height variations of ≈4 nm (Figure 3A). In contrast, the protein-coated PDMS surfaces were rougher, with height differences of >40 nm (Figure 3B).

To verify that the surfaces were coated with proteins, we tried to physically remove the protein coating over a 10 × 10 µm^2^ area of the sample by applying a high contact force (≈95 nN) during contact-mode AFM imaging, that is, by scratching the coating with the AFM cantilever. An area was scratched several times before the scanning angle was rotated 90° and the same area was scratched several more times. After scratching, a 20 × 20 µm^2^ area surrounding the scratched area was imaged. On the glass surface, the protein was removed from the scratched area (Figure 3A’’). The protein-coating depth was 3.2 ± 0.5 nm (*n* = 5) for BSA and 4.5 ± 0.4 nm (*n* = 5) for collagen I (Figure 3C). The AFM images of the PDMS coatings (Figure 3B,D) revealed an uneven surface with features exceeding 40 nm in height. These are replications of imperfections in the aluminum mold. Although the sensitivity of AFM imaging is high enough to detect protein coatings, the overall roughness of the surface conceals the thin protein layers, and, thus, detection of the presence of a scratched protein patch was not possible.

Since we were unable to confirm a protein coating on PDMS by the AFM scratching experiments, we coated the glass and PDMS surfaces with fluorescent-conjugated proteins used in the later adhesion study: fluorescein isothiocyanate (FITC) conjugated BSA, rhodamine-conjugated laminin, FITC-conjugated collagen I and HiLyte488-conjugated fibronectin. For each protein, confocal images show nearly homogeneous surface signals when absorbed on both PDMS (Figure 3E’–H’) and glass (Figure 3E–H). In a smaller area the fluorescence was bleached using an excitation wavelength of either 488 nm or 555 nm. On both surfaces the fluorescence signal was quenched, demonstrating that the ECM proteins did in fact coat the surfaces. Negative controls (uncoated glass or PDMS) showed background fluorescence that did not decrease with bleaching (data not shown). These experiments confirm homogeneous protein coatings on glass surfaces and indicate that the PDMS surfaces were similarly coated.

### Accessibility of the coated area with cantilever-bound cells

The accessible area at the bottom of a SCFS well is limited by the geometry of the mask and the AFM cantilever. If these come into contact with each other, the recorded force–distance curves will be corrupted or, worse, the AFM cantilever may be displaced and the cantilever can be damaged. Both the height of the coating mask and the 10° angle of the cantilever determine the accessible area. Unfortunately, PDMS masks thinner than 150 µm are fragile and difficult to handle, making the production of masked-dishes cumbersome. The area suitable for adhesion measurements is depicted in Figure 2C. Since the AFM chip must clear the chip-side of the mask, an approximately 4 mm^2^ area at the chip-side cannot be used for adhesion measurements (see Figure 2C, white area). How close the cantilever can be moved towards the side borders of the well is determined by the position of the cantilever on the chip and how the chip is mounted. The tip-side area is accessible until the cantilever makes contact with the mask. When the cantilever was too close to the chip boarder, both approach- and retraction-force–distance curves showed a distinct bending in the baseline force. When the cantilever was too close to a side border, no obvious features were found in force–distance curves. The sensitivity of the cantilever was determined by pressing it onto a surface either outside the mask or close to the tip-side border of a well.

### Evaluation of the PDMS masks for light microscopy

Light microscopy is essential for many SCFS experiments. Therefore, we tested the optical behavior of the masks with PDMS surfaces. UV–vis spectra of 1 mm thick PDMS slices showed that cured PDMS did not absorb light at wavelengths important for light microscopy (230–840 nm, data not shown). PDMS masks did not appear to reduce the quality of wide field and fluorescence images. However, the *z*-resolution and calibration will be suboptimal since the refractive index of PDMS (1.415, as measured) does not match that of water. Furthermore, the working distance of the microscope objective must exceed the thickness of the PDMS layer, thus, these masks are not suitable for short distance, high numerical aperture objectives. Although the masks with PDMS surfaces show technical limitations in optical microscopy, they are usable for standard microscopy mostly used in combination with SCFS. If the PDMS masks interfere with optical microscopy, the masks with glass surfaces are recommended.

### Comparison of the non-specific adhesion force on glass and PDMS surfaces

**Figure 4:**
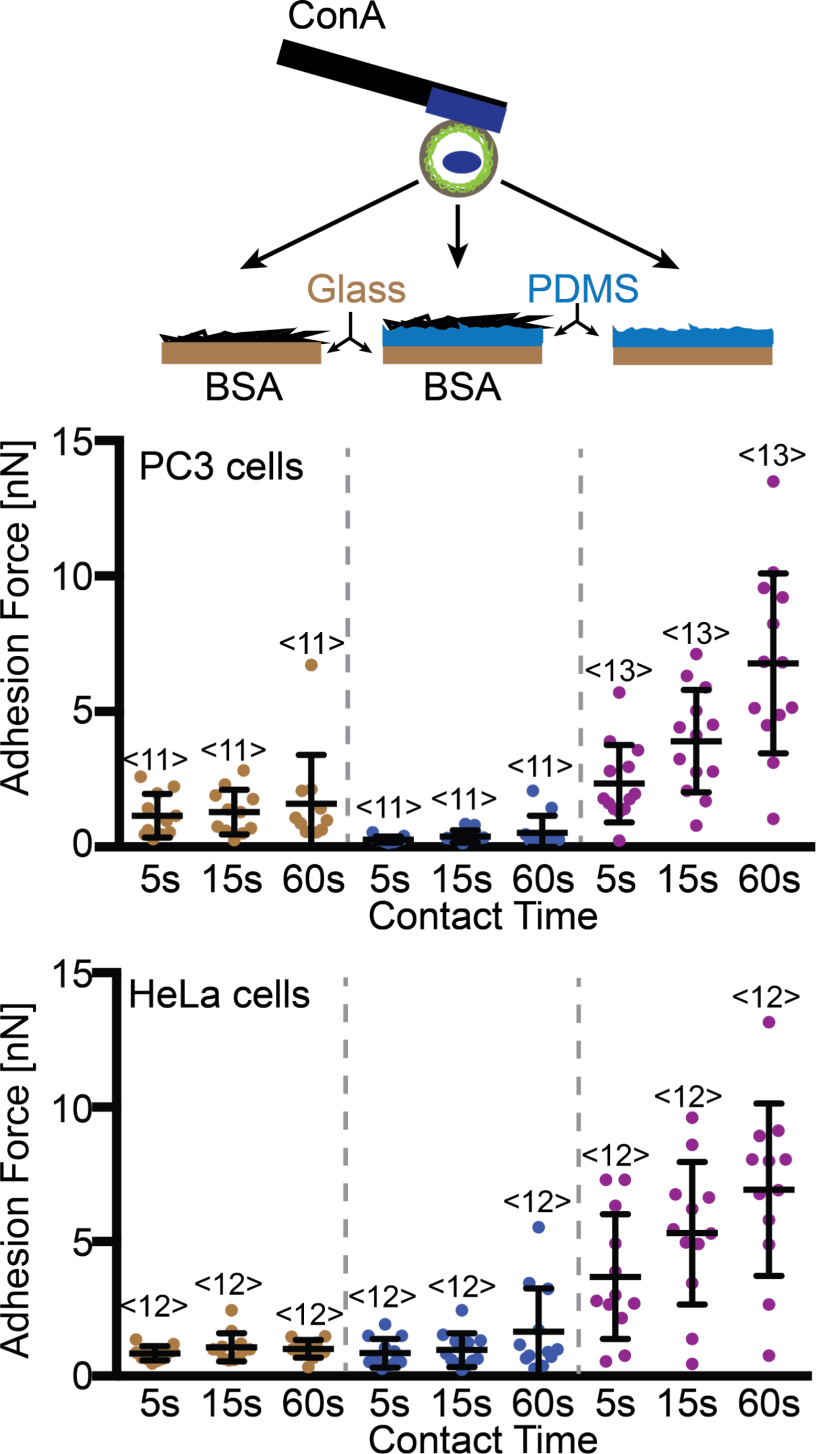
Comparisons of glass-surface and PDMS-surface masks**.** Top, depiction of the SCFS assay used to quantify the adhesion of PC3 and HeLa cells. Single cells were bound to ConA-coated cantilevers and approached BSA-coated glass or PDMS and a clean PDMS surface until a force of 2 nN was recorded. After the denoted contact time, the cantilever was retracted to detach the cantilever-bound cell and substrate. During retraction, the adhesion force of cell and substrate was measured. Bottom: adhesion forces recorded for PC3 and HeLa cells during their detachment from substrates. Each dot represents the measurement of one cell with the number of cells assayed for each condition given by <*n*> The *x*-axis indicates the duration (5, 15 and 60 s) that the cell was in contact with the substrate before being detached. The error bars indicate mean force and standard deviation.

In our experience, non-specific cell adhesion to clean glass is higher than the adhesion of cells to ConA-coated cantilevers. In order to attach cells to ConA-coated cantilevers, it was necessary to passivate glass surfaces onto which cells were pipetted. We found that both HeLa and PC3 cells also strongly adhere to PDMS (Figure 4). Therefore, it was necessary to passivate the PDMS surfaces. BSA is commonly used to block non-specific adhesion of cells to different surfaces [38]. To address non-specific cell adhesion, we coated glass and PDMS wells with BSA. To our surprise, the adhesion of PC3 cells was higher for BSA when BSA was adsorbed onto glass than when absorbed onto PDMS (Figure 4). Thus, we decided to use PDMS masks in subsequent experiments. Furthermore, the cells were collected with the cantilever from BSA-coated wells because their adhesion to BSA is lower than to the ConA-coated cantilevers.

### Cell line-dependent adhesion to extracellular matrix proteins

**Figure 5:**
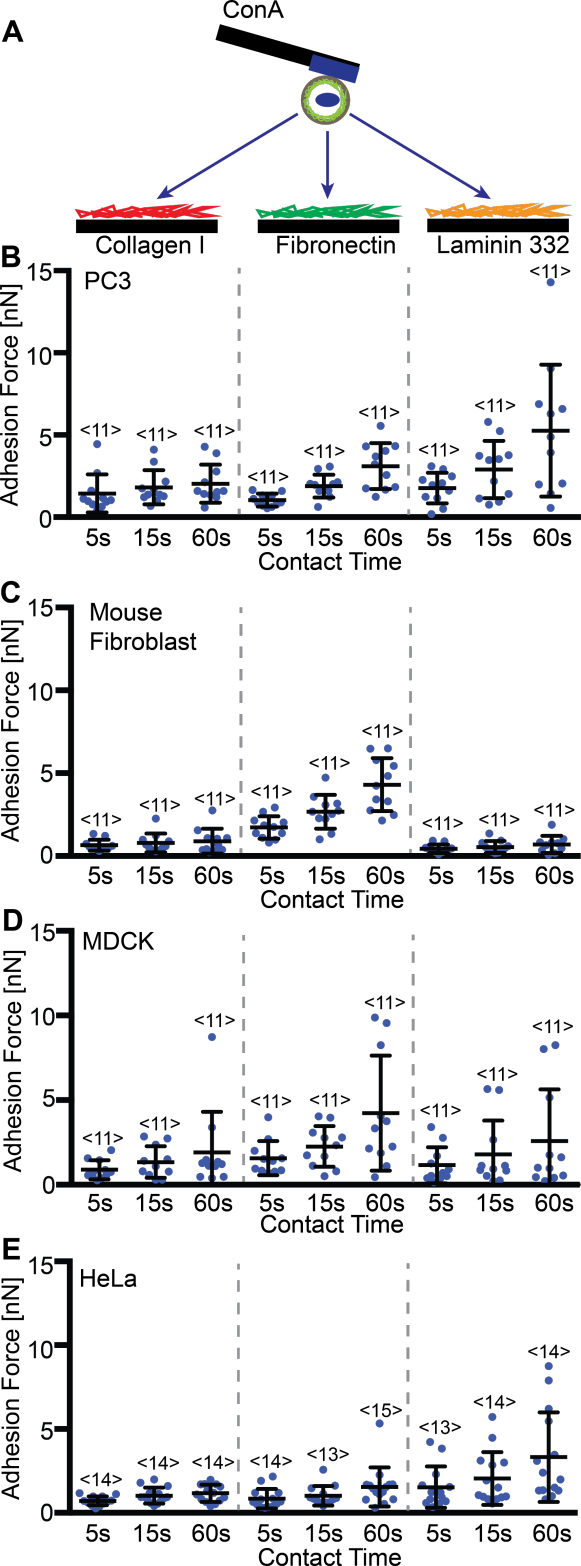
Cell line-dependent adhesion of ECM proteins. (A) Depiction of the SCFS experimental setup, where the adhesion of a ConA bound cell is measured to different protein-coated surfaces. Graphs of the adhesion forces measured for PC3 (B), mouse fibroblasts (C), MDCK (D) or HeLa (E) cells to collagen I, fibronectin and laminin 332 coated PDMS surfaces after contact times of 5, 15 and 60 s. Each dot represents the measurement of one cell. The number of cells assayed for each condition is given by <*n*>. Error bars indicate mean force and standard deviation.

To demonstrate that PDMS masks are a useful tool to increase throughput and comparability of results on different ECM proteins in SCFS, we conducted a small adhesion-force screening with four cell lines and three different ECM proteins. Thereto, we coated the wells in the PDMS-coated masks with collagen I, fibronectin, and laminin 332. We evaluated cell adhesion of PC3, HeLa, mouse kidney fibroblast and MDCK cells to different extracellular matrix proteins. The fourth well of the PDMS mask was coated with BSA from which cells were picked up. We measured the adhesion force to all three ECM proteins for at least 11 cells of each cell line (Figure 5). As expected, the cell lines differed in their adhesion to the ECM proteins. For example, PC3 cells showed similar adhesion to collagen I and fibronectin and a higher adhesion to laminin 332, while mouse kidney fibroblast showed very low adhesion to collagen I and laminin 332, but high adhesion to fibronectin. This indicates that cell lines express different patterns of CAMs. Besides the cell line-dependent adhesion forces, contact time-dependent strengthening also differed between cell lines, indicating difference in the dynamic regulation of adhesion. These results show that the PDMS masks can be used to evaluate the adhesive properties that distinguish different cell lines from each other and perhaps reflect unique cell line-specific adhesion signatures.

## Discussion

Adhesion is a fundamental aspect of both healthy and diseased cells. In the last decade, single cell adhesion studies have contributed to the understanding of adhesion proteins and their regulation. AFM-based SCFS has been used to quantify adhesion of numerous cell types to a diverse set of substrates, include ECM proteins, biomaterial and cell–cell adhesion proteins [14–15,26]. However, since only one cell can be examined at a time, the number of conditions that can be studied efficiently by SCFS is limited. To help alleviate this problem, we designed two kinds of PDMS masks that allow adhesion of one cell to multiple adhesive substrates to be measured. The masks were cast in aluminum molds, which can be made in most mechanical workshops.

We characterized the masks with regard to surface topography, protein coating ability and applicability for light microscopy. While the PDMS surface was very rough compared to the glass surface, both could be well-coated with proteins. The height variation of the PDMS surface (>40 nm), although small compared the size of cells examined (diameter, 15 µm), may be the cause of the differences in HeLa cell adhesion to BSA-coated substrates observed in Figure 4. Other differences between the glass and PDMS surfaces, such as hydrophobicity or the specifics of protein absorption, may also account for this difference. However, since the difference in adhesion to BSA only occurred for PC3 cells (Figure 4) and was minor compared to specific adhesion, we conclude that both glass and PDMS surfaces can be coated and used for cell adhesion measurements. Fluorescently labeled proteins adsorbed on glass and PDMS showed homogenous protein coatings on both surfaces (Figure 3), and confirmed this conclusion. Further experiments showed that like glass-bottomed wells, PDMS-bottomed wells are suitable for light microscopy. Thus, neither mask lowered the quality of possible SCFS experiments, even in combination with light microscopy. If SCFS is combined with advanced light microscopy and objectives with short working distances and high numerical apertures are used, the glass surface masks are preferable.

Finally, we used the PDMS masks to characterize and compare the adhesion of four cell lines to collagen I, fibronectin and laminin 332. HeLa (Kyoto), PC3, mouse kidney fibroblast and MDCK cells where chosen based on their widespread use. The masks allowed us to profile the adhesion properties of the cells quickly and efficiently, requiring ≈2 days for each cell line. By measuring the adhesion of a single cell to different matrix proteins, a smaller number of cells need to be assayed. These measurements show that cell lines have specific ECM protein adhesion profiles. This is likely due to difference in their expression of integrins. To profile cell adhesion of different cell lines, the used substrates could be readily extended to more ECM proteins and cell–cell adhesion proteins, such as E-cadherin. Further, contact times could be increased to address long-term CAM regulation, which would increase the accuracy of the profiles. With the help of the PDMS-coated masks, a semi-automated adhesion measurement setup is feasible.

Our masks compare favorably to commercially available silicone masks, such as those offered by Ibidi. The production of the mask is easy and commercially available equivalents are commonly expensive. The masks described herein require a mold, which can be easily produced in workshop, and the PDMS components. The usage of the mask is not limited to AFM-based SCFS, as the four-well mask is only one of many possible dimensions and forms. For example, migration or spreading experiments on different proteins could be conducted using PDMS masks. Further examples where masks could be used include wound-healing assays and experiments that require more complex surfaces.

## Experimental

### Production of PDMS masks

To produce the PDMS masks, a casting mold with cavities for nine masks was used (Figure 2A). For the mold, 150 µm-deep cavities were machined in an 8 mm-thick aluminum plate (Figure 2B), which was then anodized to harden its surface. To reduce adhesion of PDMS to the mold, the mold was silanized overnight with 200 µL of perfluorooctyltrichlorosilane in a vacuum chamber at room temperature. After silanization, the mold was washed extensively with water and ethanol. It was important that the cavities were free of any residues before casting. The PDMS elastomer (Sylgard 184, Dow Corning) was mixed in a 10:1 oil to base ratio. The mixture was placed in a vacuum chamber for 20 min to remove dissolved gases. Approximately 2 mL of the uncured PDMS mixture was poured on the top of the aluminum mold and spread uniformly over the mold using a straightedge. For glass surface masks, only the cavities of the mold were filled and, importantly, the inner squares (future wells of the mask) were wiped free of PDMS. In contrast, to produce masks with PDMS surface wells, the entire top of aluminum mold was left covered with a thin PDMS layer. The mold was place in an oven at 80 °C for at least 2 h to fully cure the PDMS. After cooling, the glass-bottom masks were carefully lifted out of the cavities and excess PDMS removed from the edges of the wells. In contrast, PDMS-surface masks where cut out of the continuous PDMS layer before they were lifted from the mold. Subsequently, both types of masks were placed topside down in the middle of a glass bottom Petri dish (WPI) and any air bubbles were removed by gently pushing them out with tweezers before the dishes were heated at 80 °C for 20 min. To enhance the binding of the PDMS mask to the glass, both surfaces can be plasma cleaned in air for one minute before they are joined. However, PDMS surfaces become hydrophilic during plasma treatment, and thus, liquid coating drops spread over the masks. Storing the PDMS in air will restore the hydrophobicity of the surface.

### Cell culture

PC3 cells were maintained in RPMI-1640-supplemented (Gibco-Life technologies) 1 mM sodium pyruvate; HeLa cells (Kyoto) and mouse kidney fibroblasts were maintained in DMEM GlutaMAX supplemented with 10% (v/v) FCS; MDCK cells were maintained in MEM supplemented with 5% FCS. All media also contained 100 units/mL penicillin and 100 µg/mL streptomycin (Gibco-Life technologies).

### Protein functionalization of PDMS masks and the AFM cantilever

Before the PDMS or glass surface was coated with proteins, the Petri dish containing the PDMS masks was washed with ethanol and ultrapure water to remove any residue. After drying the Petri dishes, 16 µL solutions of 160 µg/mL collagen I (Inamed Biomaterials), 50 µg/mL fibronectin (Merck), 50 µg/mL laminin 332 (Abcam) or 2% (w/v) BSA (Sigma) in PBS were added to the separate wells and left to adsorb overnight at 4 °C. In order to minimize uncoated glass areas, all wells were incubated with 2% BSA in PBS for 30 min at room temperature. The glass and PDMS were coated with fluorescently labeled proteins as described for non-labeled proteins. The cantilever coating was performed as previously described [30]. In short, the cantilevers were plasma-cleaned and incubated overnight in 2 mg/mL ConA (Sigma) containing PBS at 4 °C.

### Characterization of protein coatings

To characterize surface roughness and protein coatings of PDMS and glass-surfaced wells, a NanoWizzard II AFM (JPK Instruments) mounted on an inverted microscope (Axio Observer.Zi, Zeiss) was used. AFM imaging was performed in intermittent contact mode with a v-shaped cantilever (SNL, Brucker) having a nominal spring constant of 0.58 N/m. First, an area of 20 × 20 µm^2^ was imaged at a line rate of 0.7 Hz and with a resolution of 512 × 512 pixels. During the scan, the force acting on the surface was kept low by manually adjusting the drive voltage between 0.5 and 1 V, which compensated for the thermal drifts. AFM scratching was done in contact mode on an area of 10 × 10 µm^2^ with a cantilever deflection of 8 V (≈95 nN), a line rate of 10 Hz and an image size of 128 × 128 pixels. After performing 10 scratches, the scan direction was rotated 90° and another 10 scratches were performed. Thereafter, the original 20 × 20 µm^2^ surface area was reimaged using the original AFM settings.

### Single-cell force spectroscopy

For SCFS, a CellHesion200 (JPK Instruments) device mounted on an inverted optical microscope (Axio Observer.Z1, Zeiss) in a temperature-controlled, noise cancellation box was used. The temperature was set to 37 °C throughout the experiments. 200 µm-long, tip-less, v-shaped, silicon nitride cantilevers having nominal spring constants of 0.06 N/m (NP-O, Bruker) were used for adhesion measurements. The spring constant of every cantilever was determined prior to the experiment using the thermal noise method.

Prior to experiments, cells grown to ≈80% confluency were detached from culture flasks by trypsin/EDTA and washed off with measurement media (cell line-specific media supplemented with 20 mM HEPES) containing 10% FCS. Cells were pelleted (420 g for 90 s) and resuspended in measurement media. Petri dishes with PDMS masks were washed with measurement media to exchange coating buffers and remove loosely bound protein from the surface. Throughout the experiments, the PDMS mask remained on Petri Dish. After a recovery time of at least 45 min in the measurement media [20], cell suspensions were pipetted onto the BSA well and cells were allowed to settle. To attach a cell, the calibrated and functionalized cantilever was lowered onto a cell at a velocity of 10 µm/s until an upward force of 5 nN was recorded and was then raised after remaining for 5 s at a constant height. The presence of a cell at the apex of the cantilever was visually confirmed. Cantilever-bound cells were incubated for 10 min to ensure firm binding on the cantilever. For adhesion measurements, single cantilever-bound cells were moved over the protein-coated wells and lowered onto the protein-coated surface with a velocity of 5 µm/s until a contact force of 2 nN was reached. The cantilever was maintained at a constant height for 5, 15 or 60 s (contact time) and subsequently retracted a distance of >90 µm at a speed of 5 µm/s until the cell was fully detached from the substrate. After each adhesion cycle, the cell was allowed to recover for a time at least equal to the contact time before a new adhesion cycle was performed. The spot at which each cell adhesion was quantified was changed with each adhesion cycle. After quantifying the cell adhesion with the three contact times, the cell was moved to another protein-coated well and adhesion measurements were repeated. To avoid possible systematic errors caused by substrate-specific cell activation or deactivation, we varied the order in which cell adhesion to the substrates was measured. After adhesion measurements were performed for all protein coatings, the cell was exchanged. The Petri dish was replaced after characterizing 4 cells on each coating. If a cell showed morphological changes during the experiments, it was discarded. Adhesion forces were extracted from force–distance curves using JPK data processing software.

### Fluorescence microscopy and UV–vis spectroscopy

Fluorescence microscopy was performed with an inverted scanning confocal microscope (Axio Observer.Z1, LSM 700, Zeiss) equipped with a plan, apochromat, 25×, 0.8 numerical aperture, water-immersion objective lens (Zeiss). FITC–albumin (Sigma), fibronectin–HiLyte488 (LuBioScience), laminin–rhodamine (LuBioScience) and FITC-collagen I (Sigma) were dissolved at a concentration of 20 µg/mL in PBS and absorbed as described above. For fluorophore bleaching, the laser power of the 488 or 555 nm laser was set to the maximum intensity. A limited area (20 × 20 µm^2^) was bleached using a single 156 × 156 pixel scan, before a 50 × 50 µm^2^ image (448 × 448 pixels) was recorded at 4% laser intensity. UV–vis spectra were acquired using a NanoDrop 2000c (Thermo Scientific) for 1 mm-thick PDMS slices.
